# Molecular Biomechanics Controls Protein Mixing and Segregation in Adherent Membranes

**DOI:** 10.3390/ijms22073699

**Published:** 2021-04-02

**Authors:** Long Li, Bernd Henning Stumpf, Ana-Sunčana Smith

**Affiliations:** 1 PULS Group, Institute for Theoretical Physics and Interdisciplinary Center for Nanostructured Films, Friedrich-Alexander-University Erlangen-Nürnberg, Cauerstrasse 3, 91058 Erlangen, Germany; long.li@fau.de (L.L.); henning.stumpf@fau.de (B.H.S.); 2 Key Laboratory of Mechanics on Disaster and Environment in Western China, Ministry of Education, College of Civil Engineering and Mechanics, Lanzhou University, Lanzhou 730000, China; 3 Group for Computational Life Sciences, Division of Physical Chemistry, Ruđer Bošković Institute, Bijenička cesta, 10000 Zagreb, Croatia

**Keywords:** membrane adhesion, protein biomechanics, phase space, nanodomain, receptor-ligand pairs, adhesive contact

## Abstract

Cells interact with their environment by forming complex structures involving a multitude of proteins within assemblies in the plasma membrane. Despite the omnipresence of these assemblies, a number of questions about the correlations between the organisation of domains and the biomechanical properties of the involved proteins, namely their length, flexibility and affinity, as well as about the coupling to the elastic, fluctuating membrane, remain open. Here we address these issues by developing an effective Kinetic Monte Carlo simulation to model membrane adhesion. We apply this model to a typical experiment in which a cell binds to a functionalized solid supported bilayer and use two ligand-receptor pairs to study these couplings. We find that differences in affinity and length of proteins forming adhesive contacts result in several characteristic features in the calculated phase diagrams. One such feature is mixed states occurring even with proteins with length differences of 10 nm. Another feature are stable nanodomains with segregated proteins appearing on time scales of cell experiments, and for biologically relevant parameters. Furthermore, we show that macroscopic ring-like patterns can spontaneously form as a consequence of emergent protein fluxes. The capacity to form domains is captured by an order parameter that is founded on the virial coefficients for the membrane mediated interactions between bonds, which allow us to collapse all the data. These findings show that taking into account the role of the membrane allows us to recover a number of experimentally observed patterns. This is an important perspective in the context of explicit biological systems, which can now be studied in significant detail.

## 1. Introduction

Cell adhesion is a complex biological process involving a variety of receptors and their ligands of different length, flexibility and affinity [1] and is involved in numerous cellular processes [2]. Many physical aspects of cell adhesion were elucidated using mimetic models that offer full control of the system composition and experimental conditions [3,4]. For example, quantitative understanding obtained from the combined experimental and theoretical studies of adhesions of giant unilamellar vesicles (GUVs) could be used to rationalize the behavior of cells [3,5,6] in the context of the role of binder density [7] and mobility [7,8], or patterning [9,10]. One highlight of the work on mimetic systems is the establishment of a link between the properties of the environment of a flexible fluctuating membrane and the reaction rates for the formation of bonds, which affects the nucleation and growth of adhesion domains [11]. Nevertheless, so far, most of the theoretical and experimental mimetic cell work focused on the formation of adhesion domains consisting of only one protein pair [11], or mixtures between binders and repellers [12,13,14].

In many cells, however, several bond types act simultaneously. Characteristic examples are the activation of Natural Killer cells [15,16] or T Cells [17] which depends on the integration of activating and inhibitory signals [16], or the rolling of the leukocytes over the endothelial cells which is mediated by the interplay of the selectin and integrin binding [18]. Despite such biological significance, there are only scarce attempts to understand the interplay of multiple binding pairs differing by their mechanical and biochemical properties [19,20]. Initial works suggested that, if protein-bridges differ in length, they will separate into two macroscopic adhesion domains, each containing only one bridge type [19]. On short time scales, more complex transient adhesion domains were found dependent on the diffusive properties of binders [21], while complex structures, including bullseyes pattern characteristic in adherent T-Cells, required the introduction of active forces [20]. Despite these efforts, small stable domains, or even domains containing more than one type of bridge have not been reported in mimetic systems so far, although commonly appearing in the cellular context. We hypothesize that this type of organization relies on membrane mediated interactions which were found crucial for recovering the microscopic and macroscopic patterns in single ligand-receptor pair studies [9,11].

**Figure 1:**
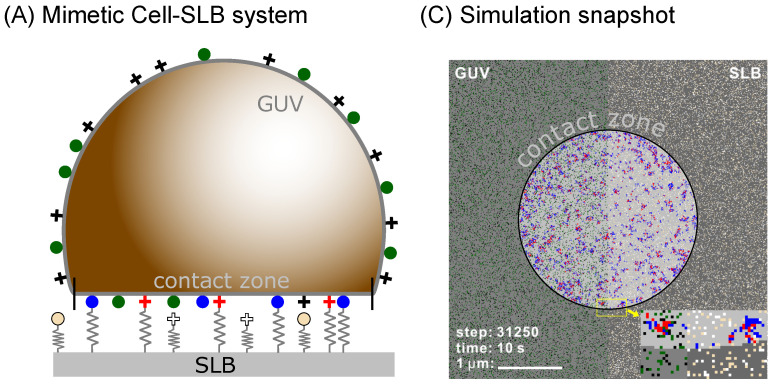

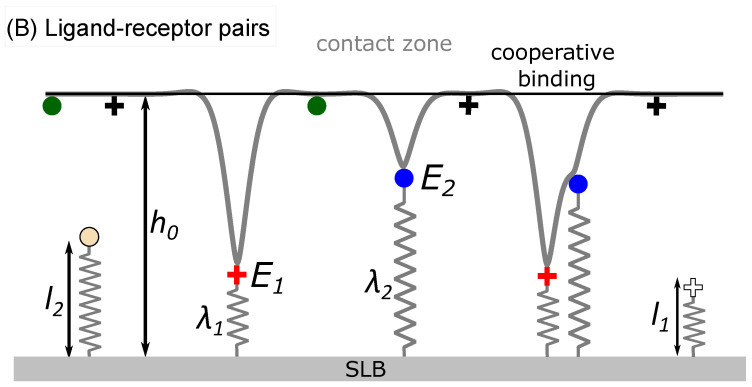
(**A**) Upon spreading, the mimetic cell establishes a contact zone at the distance $h_{0}$ of 50–100 nanometers from the functionalized solid support, due to the presence of a weak potential produced by various nonspecific interactions. (**B**) The formation of bonds deforms the membrane. To model these effects, receptors of size $l_{1},l_{2}$ are modelled as elastic springs of stiffness $\lambda_{1},\lambda_{2}$. The bond energies are denoted with $E_{1}$ and $E_{2}$. Membrane deformations can be shared between nearby bonds, increasing the binding chances. (**C**) The bonds can statistically form and break following coarse grained reaction rates that take into account the correlations between bonds and the mechanical state of the membrane [22]. Depending on the strength of the correlations, the bonds will form different types of domains. In the snapshot from the simulations, the two types of bonds can form only in the central circular domains and are shown in blue and red. On the left part of the frame, the receptors on the mimetic cell are shown with green and black, while on the right half, the ligands on the support are shown with yellow and white dots.

To test this hypothesis, we develop a simulation framework (see Methods section below) for studying systems with multiple binder types as shown in Figure 1. We implicitly account for the effects of the membrane, and are able to study the pattern formation on time scales of microseconds to hours, in a system which has the size of a cell, yet resolving each protein. We use this model to test binary mixtures of ligand-receptor pairs to study the steady state organization of bonds as a function of the lengths and intrinsic binding strength of the contributing proteins. As a result, we find a rich phase space with diverse morphologies of adhesion domains, including small yet dense domains containing only one type of bond as well as fully mixed domains where the two types readily coexist.

## 2. Effect of the Difference in the Length of Ligand-Receptor Pairs

We first explore the organization of ligand-receptor constructs of two different lengths that are forming in the contact zone. Both receptor types are equally stiff ($\lambda_{i}=\lambda_{1}=\lambda_{2}$) and bind to ligands with the same intrinsic affinity ($E=E_{1}=E_{2}$). In both cases, the binding takes place within a pocket of a size α.

**Figure 2:**
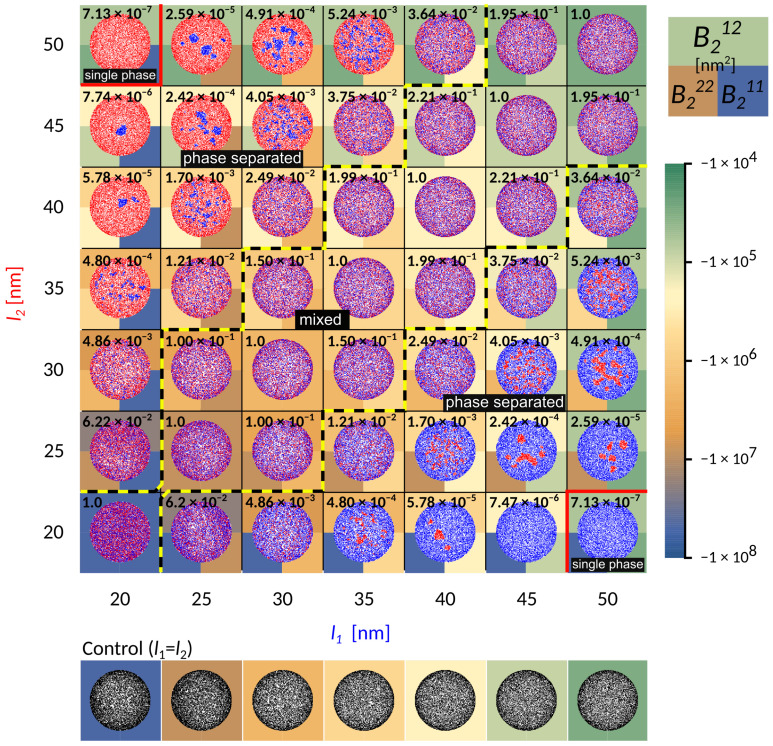
Contact zone for simulations with two types of ligand-receptor bonds of varying lengths as indicated on the axis, shown in red and blue. The axis labels are colored according to the bond type. Free binders are omitted for clarity. Besides the length, other characteristics of the bonds are identical and chosen to be $E_{1}=E_{2}=7k_{B}T$, $\lambda_{1}=\lambda_{2}=5\times 10^{4}k_{B}T/\mu m^{2}$, $h_{0}=60\mathrm{nm}$. The other parameters are as indicated in the table in the Methods section. The background color stands for the calculated second virial coefficient, which is a measure of attraction between bonds of same or different type. The top half of the square is coloured according to the magnitude of the virial obtained from the potential between bonds of different type, while the bottom left and right squares are values of the virials from the potentials of interactions acting between two identical bonds of type 1 and 2, respectively. The number indicated in the top left corner is the value of the order parameter ξ in the relevant system.

To represent relevant biophysical situations, the system parameters (Table 1 in the Methods) are chosen such that irrespective of the length, adhesion by a single bond-type fills up the contact zone without forming a single, tightly packed structure (see control simulations in Figure 2). With this particular choice of conditions, the formation of dense domains, consequently arises from the presence of receptors of different lengths.

When the receptors are of similar length (the diagonal in Figure 2), the two bond types are mixing already on the scale of first neighbors. Interestingly, the co-localisation of the two different adhesive constructs persists for differences in protein length of up to 10 nm (area of the phase space between the yellow-black lines marked as “mixed”). This state is enabled by a complete lack of direct lateral interactions (cis interactions) between binders, which would promote oligomerisation even in solution. Notably, for shorter receptors close to $l_{0}=20\mathrm{nm}$, these mixed states form from radially spreading domains (bottom left part of the phase diagram). On the other hand, for smaller affinities and smaller separations, adhesion proceeds through the formation of numerous and small, but mixed clusters that quickly merge (top right part of the phase diagram). The composition of the mixed state depends on the relative densities of the two ligands and receptors, as discussed recently [23].

This behaviour is the consequence of the membrane mediated interactions between bonds $V_{B}^{12}\left(r\right)$, at the distance $r=|r_{1}-r_{2}|$, with $r_{1}$ and $r_{2}$ denoting the spatial coordinates of bond of a type 1 and 2, as indicated in the superscript. This interaction between two bonds can be calculated following previous work [24,25] under the condition that the potential vanishes at r→∞. Consequently the interaction energy between two mechanically distinct bonds reads

(1) $$\begin{array}{c} V_{B}^{12}\left(r\right)=\frac{4\lambda_{1}\lambda_{2}\lambda_{m}\mathrm{kei}\left(q_{0}r\right)}{\pi^{2}\left(\lambda_{m}^{2}+\lambda_{1}\lambda_{2}+\lambda_{1}\lambda_{m}+\lambda_{2}\lambda_{m}\right)-16\lambda_{1}\lambda_{2}\mathrm{kei}\left(q_{0}r\right)}\;\cdot \;\\ \left(\pi \Delta l_{1}\Delta l_{2}+\left(\frac{2\Delta l_{1}^{2}\lambda_{1}}{\lambda_{1}+\lambda_{m}}+\frac{2\Delta l_{2}^{2}\lambda_{2}}{\lambda_{2}+\lambda_{m}}\right)\mathrm{kei}\left(q_{0}r\right)\right)+\frac{1}{2}\log\left(1-\frac{16\lambda_{1}\lambda_{2}\mathrm{kei}{\left(q_{0}r\right)}^{2}}{\pi^{2}\left(\lambda_{1}+\lambda_{m}\right)\left(\lambda_{2}+\lambda_{m}\right)}\right), \end{array}$$

where $\Delta l_{i}=h_{0}-l_{i}$, and $q_{0}$ is the inverse of the correlation length. In the case of a tensionless Gaussian membrane $q_{0}=1/\sqrt[{4}]{\kappa /\gamma }$, and $\lambda_{m}=8\sqrt{\kappa \gamma }$. For membranes with tension, a similar expression can be derived [25]). However, for active membranes, the relation of the correlation length and the membrane effective stiffness with tension, activity, stiffness and other mesoscocpic properties are not yet established. Nonetheless, as long as the membrane shape fluctuations are fast compared to the bond dynamics, the Equation (1) may be applied. In our calculations, besides accounting for the two-bond interaction potential, Equation (1), we account for the hard core repulsion between the proteins, which prevents them to be at the same lattice site. Consequently the total potential is modified by a hard core repulsion.

(2) $$V_{2}^{12}\left(r\right)=\left\{\begin{array}{cc} \infty & r\leq a\\ V_{B}^{12}\left(r\right) & r>a \end{array}\right.$$

**Figure 3:**
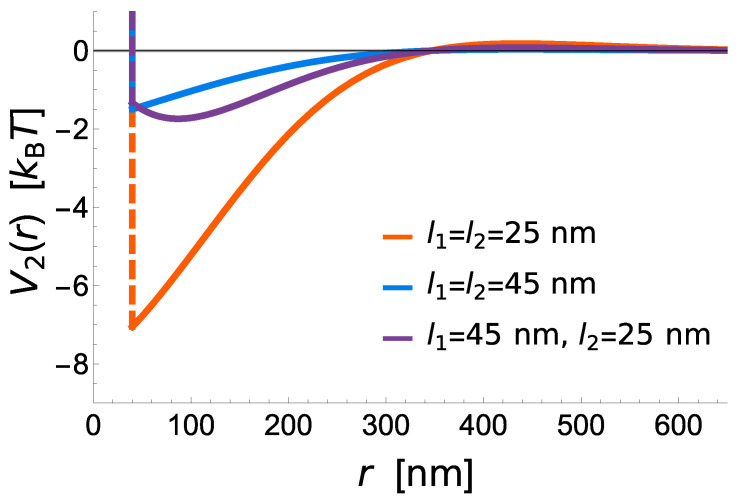
Interaction potentials $V_{2}^{12}$ between two bonds of varying lengths and $\lambda_{1}=\lambda_{2}=5\times 10^{4}\;k_{B}T/\mu m^{2}$. Asymmetric bonds with unequal rest lengths show an additional repulsion, as the bonds work against each other in trying to pull the membrane to different heights. However, as the membrane height relaxes around a bond inducing strong deformations, there is a distance at which bonds inducing little membrane deformation can preferentially attach, resulting in a potential minimum that is shifted outwards compared to symmetric bonds.

The potential shows that short receptors introduce stronger membrane mediated interactions, promoting radial growth compared to long constructs, where these interactions are negligible (Figure 3). The effective binding affinity will only affect the bond stability, but has no further influence of the strength of interactions between two bonds. It is instead set by the vertical separation between the ligands and the binding sites on the protein (i.e., the protein length), the flexibility of extracellular domains (i.e., protein elastic constant), the stiffness of the membrane (bending rigidity), and the fluctuation amplitude of the membrane (Figure 3).

At larger difference in bond length ($|l_{1}-l_{2}|>10$ nm), the shorter bonds start to agglomerate into small domains throughout the contact zone (‘phase-separated’ region in Figure 2). This is clearly promoted by the presence of long bonds when the domains of short bonds minimize the penalty of bending the membrane, effectively creating an interface with the lattice gas of long bonds. While the choice of the initial conditions may affect the macroscopic details in the steady state, throughout the majority of the phase space we observe multiple stable domains, contrary to previous theoretical reports but in agreement with experiments [14]. This suggests that the line tension building up at the edge of the domain [20], is not sufficient to drive the full segregation against the mixing entropy in the system. Actually, the domains remain stable with ligand-receptor constructs forming a cooperative state, where membrane mediated forces effectively stabilize the short bonds. However, as the line tension increases with the strength of the interaction potential and the difference between bond lengths, the domains coarsen. Eventually, the difference in length between long and short bonds becomes so large that the short one can no longer nucleate ($|l_{1}-l_{2}|>30$ nm), and a single phase appears in the equilibrium. In this regime, the long bonds effectively act as repelling agents for short constructs, since in their absence, short bonds would normally form. This coarsening process can be clearly observed by focusing on a single column or row in Figure 2.

To quantitatively explore the protein segregation in the adherent membranes, we calculate the second virial coefficient for two-dimensional potentials $B_{2}^{XY}$, with X and Y denoting the first and the second bond-type

(3) $$B_{2}^{XY}\left(T\right)=\pi \int_{0}^{\infty }\left(1-\exp\left(-\frac{V_{2}^{XY}\left(r\right)}{k_{B}T}\right)\right)r\mathrm{dr}.$$

The virial (see the background colour in Figure 2) defines a relative interaction strength, which takes the potential over the whole range of distances into account, and is typically negative for attractive interactions, and positive for repulsive interactions.

Notably, as the length of the bond changes from 50 nm to 20 nm, and the separation from the ligand increases from 10 nm to 40 nm, the attraction of the potential increases over four orders of magnitude. While this indicates that these interactions are indeed strong and important, we find, however, that it is not the value of the virial between two bonds that captures the phase behaviour. To account for the transition between mixed and segregated states we construct a dimensionless order parameter ξ, which we defined as

(4) $$\xi =\frac{B_{2}^{sl}}{B_{2}^{ss}}\frac{B_{2}^{ll}}{B_{2}^{ss}}$$

Here, the superscripts denote a the virial coefficients for two different (sl) or identical (ll and ss) bonds, with $B_{2}^{ll}<B_{2}^{ss}$. The value of the order parameter has been indicated explicitly as a number in the background of each simulation frame in Figure 2.

**Figure 4:**
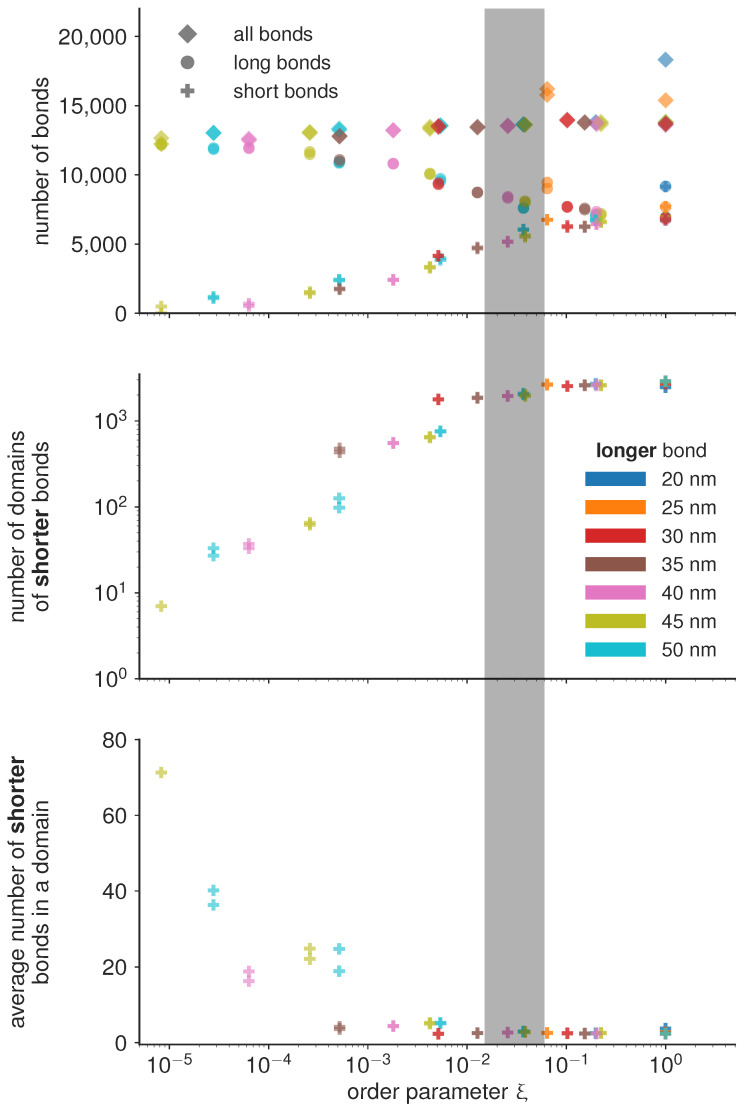
Quantification of the number of bonds and domains for the final frame of the simulation, as a function of the order parameter as defined in Equation (4) and colored according to the length of the longer bond. The according final frames are shown in Figure 2. While the total number of bonds is roughly constant, there is a transition in the phase behaviour, marked by the shaded overlay, where the shorter bond starts to form larger isolated domains compared to the gas like behaviour at higher values of the order parameter.

If the magnitude of attraction between short and long bonds, as well as the attraction between two long bonds is comparable to the attraction between two short bonds ξ≈1, then the the two bond types mix. Consequently, the characteristic length scale at which the two bond types are separated becomes very small, and the number of domains becomes of the same order of magnitude as the total number of bonds (Figure 4). On the other hand, if the attraction between short bonds is significantly larger than the attraction between long bonds or the attraction between the short and the long bond, than ξ≪1, and the two bond types segregate in well separated domains. The critical value of the order parameter at which this takes place is found empirically at $\xi ∼0.5\times 10^{-2}$ (denoted as the transition region with a gray bar in Figure 4).

Under the condition that all simulations are performed with the same total concentration of proteins (type 1 and type 2), we find that the number of domains in the system follow the same trends irrespective of the actual protein length, only dependent on the value of the order parameter. Similar trends are observed for the number of bonds per domain, and even for the total number of bonds of each type. In the de-mixed state, increasing the difference in bond lengths, however, systematically results in decreasing number of bonds which get distributed in a smaller and smaller number of domains (Figure 4). This result points to an interesting mechanism for limiting the number and size of adhesion sites in the contact zone. Namely, it is often considered that the total number of receptors on the cell membrane limits the size and the number of adhesions. The data presented here shows that the number of bonds can be limited before exhausting the reservoir, by introducing competitive binders. At this stage, we can hypothesize that the slope of the decay of the number and size of domains will depend on the overall concentrations of receptors and ligands, and their thermodynamic ensemble (constant number of the mimic cell, or the constant concentration in support), and the affinities of the bonds.

## 3. Effect of the Intrinsic Binding Affinities

**Figure 5:**
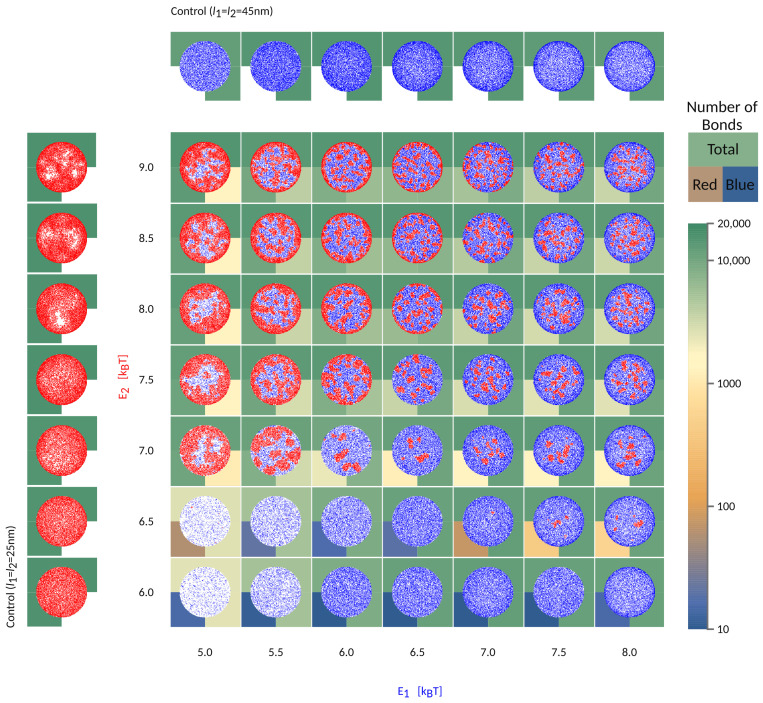
Characteristic organisation of the contact zone as a function of the binding affinities of $l_{1}=45\mathrm{nm}$ long (blue) and $l_{2}=25\mathrm{nm}$ short (red) bonds. The axis labels are colored according to the bond type. Free binders are omitted for clarity. Besides affinity, which is indicated on the axis, the other bond parameters are $\lambda_{1}=\lambda_{2}=5\times 10^{4}k_{B}T/\mu m^{2}$, $h_{0}=60$ nm. The number of formed bonds are indicated in the background of each frame using the displayed color-code. The top half of the square indicates the total number of bonds, while the bottom left and bottom right quarters are colored following the number of short and long bonds, respectively.

To further understand the segregation of bonds into domains in the contact zone, we focus on a system where $l_{1}=25$ nm $l_{2}=45$ nm, but start varying systematically the intrinsic strength of binding of the two pairs, as shown in Figure 5.

If the short bonds (red in Figure 5) are associated with a low binding affinity, their formation will be fully impeded by long bonds (blue), irrespective of the affinity of the latter ($E_{2}<6\;k_{B}T$ in Figure 5). At the same time, the formation of long bonds is significantly slowed down by the presence of the short receptors and their partner ligands, both of which are gradually expelled from the area of contact. Even in the long time limit, however, the density of long bonds is significantly smaller compared to the density in the system where the short receptors are not present (see control simulations with only one protein type in the left/top row ). This is a result of the competition for space between various species in the contact zone and the associated mixing entropy. Surprisingly, scarce domains of short weak bonds reappear when the intrinsic affinity of long bonds is comparatively larger ($E_{2}=6.5\;k_{B}T$, $E_{1}>7\;k_{B}T$). This is because the long bonds form a ring around the contact zone, preventing the evacuation of the shorter receptors, which then are able to segregate into small domains.

When the effective affinities of the two pairs are comparable, the small domains will form uniformly throughout the contact zones, including the edge. For the choice of parameters used in Figure 5, this regime takes place in the top right corner of the phase space. Notably, for larger length differences, a similar regime exists for a different ratio in $E_{1}$ and $E_{2}$, and would be set by the effective affinities of short and long bonds within their domains.

If the affinity of one bond type starts to dominate, then a macroscopic radial pattern appears in the contact zone (upper left and bottom right in the phase space). This happens because ligands and receptors with greater effective binding affinity, i.e., with shorter mean free time prior to attachment, make bonds closer to the edge, as soon as they enter the contact zone. The pair with smaller affinity, hence a longer mean free time, penetrate deeper toward the center of the contact area. As a result, we find both patterns with short (above the diagonal in Figure 2) and long bonds (bellow the diagonal) on the outer rim. These macroscopic patterns are in principle jammed structures, since the density of bonds at the edge can be so high as to practically seal the contact zone from additional recruitment. Production of these patterns and fully jammed structures are enhanced by adhesion of binding pairs that are both mobile, allowing recruitment of binders and higher enrichment ratios, compared to systems with only one mobile fraction. Furthermore, the patterning is enhanced by the very setup of the experiment. Namely, the accumulation of bonds occurs after the contact area between the cell and the bilayer has formed and because the latter acts as inexhaustible reservoirs of binders [9]. While similar effects would be seen for adhesion between two cells, the thickness of the ring would be smaller, because of the limited number of receptors on the surfaces of the two cells.

When the two pairs have similar 3D affinities, the result is that longer bonds, with greater ectodomains, settle at the periphery of the contact zone, while shorter pairs arrive closer to center, simply because the effective affinity of the longer pair dominates. These patterns resemble the basic organization of the immune synapse [19,26,27,28], where the longer ICAM-1−LFA-1 ($l_{1}∼42\;nm$ with $l_{\mathrm{ICAM}-1}=20\;nm$, $l_{\mathrm{LFA}-1}=22\;nm$) [29] surround the domains of shorter TCR−pMHC complex ($l_{2}∼14\;nm$ with $l_{\mathrm{pMHC}}=7\;nm$, $l_{\mathrm{TCR}}=7\;nm$) [29]. As shown in Figure 2 and Figure 5, the difference in length of 28 nm is sufficient to drive the separation of the two bond types and create the bullseyes like pattern, in a manner that is not very sensitive to the intrinsic binding strengths of the proteins.

**Figure 6:**
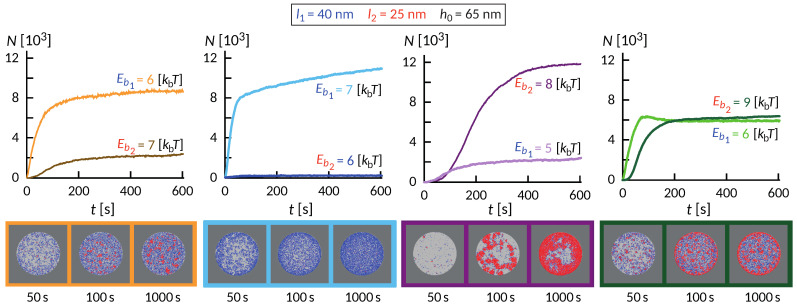
Evolution of the number of bonds with time for different binding energies. Bond lengths, $l_{1}=40\;nm$ and $l_{2}=25\;\mathrm{nm}$, and elastic moduli, $\lambda_{1}=\lambda_{2}=5\times 10^{4}k_{B}T/\mu m^{2}$, and initial separation, $h_{0}=65\;nm$, are fixed. The associated snapshots of the contact zone at t=50 s,100 s and 1000 s are displayed in the bottom sequence.

The wealth of morphology observed in the phase diagram in Figure 5 suggest a rich dynamic behavior that precedes the establishment of the steady state, as exemplified in Figure 6. Interestingly, the dynamics of the formation of these small domains is quite complex and depends on the initial size of the contact zone, spreading velocity of the contact zone and the density of the long proteins among other factors. In some cases of relatively weak bonds, the domains initially form throughout the contact zone and over time migrate toward the center or detach. This coarsening process occurs under the lateral pressure of long bonds that are recruited from the outside of the contact zone. The domains in the center, typically more than one, grow at the expense of the domains in the rim and remain stable as long as we could extend the simulation.

Despite significant differences, several common features are observed. Similar to systems with only one ligand-receptor pair, the fast initial growth is followed by a slow saturation, which can be significantly extended due to the slow influx of binders from the bulk of the SLB. This is particularly acute in ring-forming systems, where the thickness of the ring very slowly increases over time. On short timescales, an interesting interplay between the formation of short and long bonds takes place. Actually, the long bonds can both facilitate the formation of short ones, and also can impede the process, which is a non-trivial effect of membrane-mediated correlations.

## 4. Conclusions

The aim of the work presented here was to provide a general biophysical foundation and tools for exploring the effects of competition between multiple binding pairs. To this end, we presented a simulation protocol for studying cell adhesion with a binary mixture of ligands and receptors. The characteristic feature of our approach is an accurate description of membrane mediated correlations between bonds, which were previously shown to be the key for successful modeling of adhesion mediated by a single ligand-receptor pair [11].

To understand the interplay between the multiple binding species interacting simultaneously, we focus on mimicking a typical experiment in which a cell or a functionalized cell or vesicle is allowed to adhere to a supported lipid bilayer. To clearly identify the role of the biomechanical interplay between various protein constructs and the membrane, ligands and receptors are allowed to diffuse, and no *cis* interactions are introduced in the system. The key observation is the segregation of different ligand-receptor constructs, which is not driven only by the difference in the respective lengths of the bonds, but also by the correlation length of the composite cell membrane. Together, these parameters can be combined into effective affinities, the difference of which needs to be relatively significant to induce segregation of the two protein constructs [11]. All other parameters, including the intrinsic bond strength or protein densities, as well as the initial geometry of the contact area available for adhesion and the spreading velocity, determine the observed macroscopic patterns. The result is a very diverse phase space with separated and mixed adhesion domains, the latter appearing even at differences in length between two pairs of 10 nm, if intrinsic affinities are comparable.

While we focused on analyzing the phase behaviour in the adhesion equilibrium or steady states, in the long time limit, the current work suggest that interesting phenomena could be expected on much shorter time scales. However, studying nucleation dynamics requires a different type of theoretical analysis and while currently beyond the scope of this manuscript, it is surely of major interest in the context of transient adhesion in numerous cellular systems. Furthermore, given the multidimensional nature of the phase space, it is very difficult to systematically vary all system parameters. However, since the rates, the virials and the effective affinity of the bond are all a combination of the parameters, it is not overly difficult to see that changing one gives similar effects as changing another – for example, making the bond stiffer will enforce stronger deformations of the membrane and stronger attractions. The effects of various parameters can be clearly cast into the value of the order parameter. However, changing the stiffness or the separation between the adhesive interfaces will also affect the effective affinity, and hence the stability of the bonds, which may not change the nature of nanodomains, but may affect the probability for observing them.

The potential of our approach is best represented by the fact that we were able, using reasonable parameters in simulations, to recover a number of morphological features observed in adherent cells. These include the exclusion of long bonds from the nanodomains of shorter ones [30], or the formation of ring like patterns as seen previously in the formation of the synapse [17] notably, or the co-localisation of weak and strong bonds related to inflammation where weak P-selectin and strong ICAM-1 bonds work together [31].

The biological significance of these patterns should be further discussed in the context of explicit systems, where the conditions of the membrane can be measured. Furthermore, using active processes, the cell can modulate the properties of its membrane, including its composition, positioning and fluctuations, which in turn can affect the effective affinity of both pairs and consequently, their dynamic organization. Given the sensitivity of the system, change in activity can induce strong effects on the macroscopic patterns, and drive significant rearrangements on longer time scales. Nevertheless, one exceptional case should be mentioned, where the active behavior of cytoskeleton not only induces the membrane stiffness and stability changes [32] but also directly affects the surface receptor motion [33,34,35]. Specifically, during immune response, and in other processes including cell motility, the actin retrograde flow may directly couple to the movement of receptor clusters [34,35]. This type of coupling force on the receptor is possible to implement in the current scheme, however, further extension of the simulation code would be required for explicit treatment.

In closing, we show that the the competition for binding of different species can be a very powerful tool for the control of the dynamic nano- and macro-patterning of cellular membranes during the cell recognition process. While we were here focused on identifying the basic principles of this interplay, the versatility of our simulation protocol permits its application to different biological situations. These we can now address with considerable detail at realistic system sizes and geometries, covering the relevant time scales and interactions to yield reliable results.

## 5. Methods: Kinetic Monte Carlo Simulations

To simulate the growth of domains with multiple binding pairs, we extend a recently developed Monte Carlo scheme for modeling adhesion by a single ligand-receptor pair [22]. The original scheme was capable of modeling the surface of an entire vesicle (GUV) or a cell binding to a supported lipid bilayer (SLB), any other type of a flat substrate, or another cell (Figure 1A). Using the same setup, both the cell/vesicle as well as the SLB surfaces are represented by square lattices (edge-length *a*) where each individual protein occupies a single lattice site. The lattice constant is set by the typical size of a protein which can be few nanometers as in the case of 5–10 nm large integrins, 8–10 nm cadherins [11] or 4–5 nm neutravidins [9], but may also be significantly as in the case of about 40 nm large rhodopsins and other GPCRs, as stated in the Table 1. In the current simulations, we work with larger proteins but all the results scale with the lattice constant as described in previous work [22]. Consequently, it is relatively easy to convert data from one lattice to another.

The current implementation of the code can account for ligands, receptors, repellers, intermittent bridges (which can swap between membranes) and other protein types  [23]. Besides trans interactions, it is also possible to account for direct lateral *cis* interactions, and several protein types (mobile or arrested binders, tethers, repellers), however, at the expense of additional parametrisation. Notably, all proteins have the same footprint on the membrane. While this is surely a limitation of the model, which can be surpassed by applying multigrids, it allows for high computing efficiency.

The system evolves in time by alternate execution of diffusion and stochastic binding/unbinding steps until the thermodynamic equilibrium is reached and the concentration of adhesive immobile bridges saturates. The length of the time step Δt is set as $\Delta t=a^{2}/4D_{\max}$, where $D_{\max}$ is the largest of receptor/ligand diffusion coefficient $D_{i}$. Diffusion is modeled as a random walk on a lattice, where each binder attempts to move to a randomly chosen site of one of four nearest neighbors with an incidence adjusted by factor $D_{i}/D_{\max}$. If the site is already occupied by another binder, the jump is not executed. The binders attempt their move in a random order that is permuted in each simulation step. The statistical ensemble (constant protein number or constant chemical potential) of each binder type on each membrane is regulated independently [22]. Proteins may be also rendered immobile.

Upon binding, the ligands and receptors are rendered immobile. This choice is motivated by the previous reports which show that this is indeed the case in many systems. However, this may not be universally true, and the code would permit for releasing this constraint if being motivated by a particular biological system. Besides adding two more parameters and two more dimensions in the already complex multi-parametric phase space, taking into account the mobility of bonds comes also at an additional computational cost.

Binders in the contact zone can build intermittent bonds between the two membranes, which are formed and broken following coarse-grained kinetics [25,36]. In the case of ligand-receptor pairing, a bond is established with a certain probability when the binders find themselves on the opposing lattice sites inside the contact zone (circled area in Figure 1b). The rates for binding $K^{\mathrm{on}}$ and unbinding $K^{\mathrm{off}}$ depend on the pre-existing local configuration of bonds and are constructed in a similar fashion as previously discussed from the reaction rate $k_{0}$, the local membrane average position h(r) (Figure 1), the fluctuation amplitude σ(r) and the effective membrane stiffness $\lambda_{m}$ [11,22].

Instead of calculating the rates from the instantaneous configuration of bonds, which would be time consuming, we utilize the fact that lateral propagation of membrane deformation is strongly screened in the presence of many proteins [25]. Consequently, only bonds at the first two closest neighbors are taken into account [22]. This allows us to explore all possible configurations and calculate all relevant rates during the initiation steps of the simulation and store them in a hash table, which significantly speeds-up the simulation. As such, we can simulate systems of about 100 ${\mu m}^{2}$, with $10^{5}$ binders on each membrane, through an epoch of 1000 s and with 10 nm resolution, within hours on one regular processor core. However, the technique is limited by the size of the hash table $\Omega \propto 2p{\left(p+1\right)}^{4}$, where *p* is the number of different types of binders involved.

While setting up the simulation, the ligands are placed on the cell membrane and the two receptor types are put on the SLB in distinct concentrations (see Table 1 for simulation parameters). To mimic the finite size of the cell, only a constant number of ligands is considered. On the other hand, mobile receptors in the SLB are coupled to reservoirs of constant chemical potential, meaning that the concentrations of the two receptors on the SLB are maintained at constant values outside of the contact zone (current concentration variation for a system of 512 × 512 sites is bellow 5%). Initial distribution of both binders are uniform over their respective lattices. Most simulations presented in this manuscript start with an existing circular contact zone that is kept constant throughout the runs. In selected cases, the contact zone is grown with linear spreading velocity to mimic cell spreading. Simulations are interrupted when a steady state is achieved, or after $3\times 10^{6}$ steps unless specified otherwise. Here, $3\times 10^{6}$ is a defined cut-off time step after which the contact zone is closed by a ring of bonds and the adhesion domains may restructure very slowly, on time scales that are beyond the accessible ones in simulations [9].

The code and the training to use it is accessible upon request to the corresponding author.

## 6. Methods: The Analysis of the Domain Number, Size and Structure

The data was analyzed by a self-written implementation of the Hoshen-Kopelman algorithm in C++. This algorithm labels all distinct clusters, defined by any number of adjacent bonds of the same type, by a separate integer label, where every bond in a cluster is assigned the same label. Isolated bonds with no neighbours are considered to be a single cluster. We only consider grid cells connected by an edge to be adjacent, corner neighbours are not considered adjacent. Figure 4 was then produced using python and matplotlib. The total number of domains is the largest assigned label, the amount of bonds per cluster can be found by counting the number of entries for each label.

## Figures and Tables

**Table 1:** Symbols and values of parameters used in simulations with ligands and receptors, divided in groups that define lattice, diffusion, membrane, pinning and binding properties.

Quantity	Symbol	Value	Reference
**Mechanical properties of binders**			
Molecular elastic modulus	$\lambda_{1}$, $\lambda_{2}$	$5\times 10^{4}k_{B}T/\mu m^{2}$	[37]
Bond length	$l_{1}$, $l_{2}$	20–50 nm	[11,38]
**Biochemical properties of binders**			
Binding energy	$E_{1}$, $E_{2}$	$5k_{B}T$–$9k_{B}T$	[11,37,38]
Interaction range	$\alpha_{1}$, $\alpha_{2}$	1 nm	[22]
Intrinsic reaction rate	$k_{0}^{1}$, $k_{0}^{2}$	100/s	[37]
**Diffusive properties of binders**			
Diffusion coefficient of ligands and receptors	$D_{i}$	$1.25\;\mu m^{2}/s$	[37]
Diffusion coefficient of bonds		0	
Initial concentration of ligands	$\rho_{l1}$, $\rho_{l2}$	$109/\mu m^{2}$	
Initial concentration of receptors	$\rho_{r1}$, $\rho_{r2}$	$109/\mu m^{2}$	
**Properties of the unbound membrane**			
Initial separation	$h_{0}$	60–80 nm	[11,39]
Fluctuation amplitude	$\sigma_{0}$	7nm	[11]
**Simulation setup**			
Lattice constant	*a*	40 nm	[9]
Number of lattice sites	*N*	512×512	
Radius of contact zone	*R*	3.42 μm	
Temperature	*T*	300K	
Time step		320 μs	

## Data Availability

The data presented in this study are available from the corresponding author upon request.

## References

[1] Sackmann E., Smith A.S. (2014). Physics of cell adhesion: Some lessons from cell-mimetic systems. Soft Matter.

[2] Smith A.S. (2010). Physics challenged by cells. Nat. Phys..

[3] Smith A.S., Sackmann E. (2009). Progress in mimetic studies of cell adhesion and the mechanosensing. ChemPhysChem.

[4] Boulbitch A., Guttenberg Z., Sackmann E. (2001). Kinetics of membrane adhesion mediated by ligand–receptor interaction studied with a biomimetic system. Biophys. J..

[5] Fenz S.F., Sengupta K. (2012). Giant vesicles as cell models. Integr. Biol..

[6] Sengupta K., Smith A.S. (2018). Adhesion of Biological Membranes. Physics of Biological Membranes.

[7] Smith A.S., Sengupta K., Goennenwein S., Seifert U., Sackmann E. (2008). Force-induced growth of adhesion domains is controlled by receptor mobility. Proc. Natl. Acad. Sci. USA.

[8] Dillard P., Varma R., Sengupta K., Limozin L. (2014). Ligand-mediated friction determines morphodynamics of spreading T cells. Biophys. J..

[9] Schmidt D., Bihr T., Fenz S., Merkel R., Seifert U., Sengupta K., Smith A.S. (2015). Crowding of receptors induces ring-like adhesions in model membranes. BBA-Mol. Cell Res..

[10] Biswas K.H., Hartman K.L., Yu C.H., Harrison O.J., Song H., Smith A.W., Huang W.Y., Lin W.C., Guo Z., Padmanabhan A. (2015). E-cadherin junction formation involves an active kinetic nucleation process. Proc. Natl. Acad. Sci. USA.

[11] Fenz S.F., Bihr T., Schmidt D., Merkel R., Seifert U., Sengupta K., Smith A.S. (2017). Membrane fluctuations mediate lateral interaction between cadherin bonds. Nat. Phys..

[12] Limozin L., Sengupta K. (2007). Modulation of vesicle adhesion and spreading kinetics by hyaluronan cushions. Biophys. J..

[13] Dillard P., Pi F., Lellouch A.C., Limozin L., Sengupta K. (2016). Nano-clustering of ligands on surrogate antigen presenting cells modulates T cell membrane adhesion and organization. Integr. Biol..

[14] Schmid E.M., Bakalar M.H., Choudhuri K., Weichsel J., Ann H.S., Geissler P.L., Dustin M.L., Fletcher D.A. (2016). Size-dependent protein segregation at membrane interfaces. Nat. Phys..

[15] Long E.O., Sik Kim H., Liu D., Peterson M.E., Rajagopalan S. (2013). Controlling natural killer cell responses: Integration of signals for activation and inhibition. Annu. Rev. Immunol..

[16] Toledo E., Le Saux G., Li L., Rosengenrg M., Keidar Y., Bhingradive V., Edri A., Hadad U., Di Primo C., Buffeteau T. (2020). Molecular Scale Spatio-Chemical Control of the Activating-Inhibitory Signal Integration in NK Cells. BioRxiv.

[17] Hui E., Cheung J., Zhu J., Su X., Taylor M.J., Wallweber H.A., Sasmal D.K., Huang J., Kim J.M., Mellman I. (2017). T cell costimulatory receptor CD28 is a primary target for PD-1–mediated inhibition. Science.

[18] Ivetic A., Hoskins Green H.L., Hart S.J. (2019). L-selectin: A major regulator of leukocyte adhesion, migration and signalling. Front. Immunol..

[19] Weikl T.R., Lipowsky R. (2004). Pattern Formation during T-Cell Adhesion. Biophys. J..

[20] Weikl T.R., Asfaw M., Krobath H., Różycki B., Lipowsky R. (2009). Adhesion of membranes via receptor–ligand complexes: Domain formation, binding cooperativity, and active processes. Soft Matter.

[21] Tsourkas P.K., Longo M.L., Raychaudhuri S. (2008). Monte Carlo study of single molecule diffusion can elucidate the mechanism of B cell synapse formation. Biophys. J..

[22] Bihr T., Seifert U., Smith A.S. (2015). Multiscale approaches to protein-mediated interactions between membranesâ€”relating microscopic and macroscopic dynamics in radially growing adhesions. N. J. Phys..

[23] Li L., Kamal M.A., Stumpf H., Thibaudau F., Sengupta K., Smith A.S. (2020). Coexistence of Long and Short DNA Constructs within Adhesion Plaques. BioRxiv.

[24] Schmidt D., Bihr T., Seifert U., Smith A.S. (2012). Coexistence of dilute and densely packed domains of ligand-receptor bonds in membrane adhesion. Europhys. Lett..

[25] Janeš J.A., Stumpf H., Schmidt D., Seifert U., Smith A.S. (2019). Statistical Mechanics of an Elastically Pinned Membrane: Static Profile and Correlations. Biophys. J..

[26] Qi S.Y., Groves J.T., Chakraborty A.K. (2001). Synaptic pattern formation during cellular recognition. Proc. Natl. Acad. Sci. USA.

[27] Dharan N., Farago O. (2016). Formation of semi-dilute adhesion domains driven by weak elasticity-mediated interactions. Soft Matter.

[28] Burroughs N.J., Köhler K., Miloserdov V., Dustin M.L., van der Merwe P.A., Davis D.M. (2011). Boltzmann Energy-based Image Analysis Demonstrates that Extracellular Domain Size Differences Explain Protein Segregation at Immune Synapses. PLoS Comput. Biol..

[29] Barclay A.N., Brown M.H., Law S.K.A.K.A., McKnight A.J., Tomlinson M.G., van der Merwe P.A. (1997). The Leucocyte Antigen Factsbook.

[30] Razvag Y., Neve-Oz Y., Sajman J., Reches M., Sherman E. (2018). Nanoscale kinetic segregation of TCR and CD45 in engaged microvilli facilitates early T cell activation. Nat. Commun..

[31] Fuxe J., Lashnits E., O’Brien S., Baluk P., Tabruyn S.P., Kuhnert F., Kuo C., Thurston G., McDonald D.M. (2010). Angiopoietin/Tie2 signaling transforms capillaries into venules primed for leukocyte trafficking in airway inflammation. Am. J. Pathol..

[32] Li H., Yang J., Chu T.T., Naidu R., Lu L., Chandramohanadas R., Dao M., Karniadakis G.E. (2018). Cytoskeleton remodeling induces membrane stiffness and stability changes of maturing reticulocytes. Biophys. J..

[33] Swaminathan V., Kalappurakkal J.M., Mehta S.B., Nordenfelt P., Moore T.I., Koga N., Baker D.A., Oldenbourg R., Tani T., Mayor S. (2017). Actin retrograde flow actively aligns and orients ligand-engaged integrins in focal adhesions. Proc. Natl. Acad. Sci. USA.

[34] Kumari S., Curado S., Mayya V., Dustin M.L. (2014). T cell antigen receptor activation and actin cytoskeleton remodeling. Biochim. Biophys. Acta (BBA) Biomembr..

[35] Yi J., Wu X.S., Crites T., Hammer J.A. (2012). Actin retrograde flow and actomyosin II arc contraction drive receptor cluster dynamics at the immunological synapse in Jurkat T cells. Mol. Biol. Cell.

[36] Bihr T., Seifert U., Smith A.S. (2012). Nucleation of Ligand-Receptor Domains in Membrane Adhesion. Phys. Rev. Lett..

[37] Bihr T., Fenz S., Sackmann E., Merkel R., Seifert U., Sengupta K., Smith A.S. (2014). Association Rates of Membrane-Coupled Cell Adhesion Molecules. Biophys. J..

[38] Fenz S.F., Smith A.S., Merkel R., Sengupta K. (2011). Inter-membrane adhesion mediated by mobile linkers: Effect of receptor shortage. Soft Matter.

[39] Dejardin M.J., Hemmerle A., Sadoun A., Hamon Y., Puech P.H., Sengupta K., Limozin L. (2018). Lamellipod Reconstruction by Three-Dimensional Reflection Interference Contrast Nanoscopy (3D-RICN). Nano Lett..

